# Chloroform

**Published:** 1870-12

**Authors:** Charles A. Lee

**Affiliations:** Peekskill


### Correspondence.

### Chloroform.

DR. MINER:

Dear Sir,—In the article in your last number, entitled "The History of Anæsthesia," by Dr. Bennet. of Edinburgh, I notice a statement which is obviously incorrect; and, as it may lead to a dangerous and unnecessary use of chloroform, I think it important that it should be corrected. It is as follows:

"It should be understood, however, that the anæsthetic effect is produced by suspending consciousness, and, therefore, sensation and volition, by acting on the brain and medulla oblongata," &c.

Now, if any fact is susceptible of demonstration, it is that to annul sensibility, it is neither necessary to destroy consciousness nor volition, and hence not to effect the medulla oblongata at all.

I need not say that, when chloroform is given to this extent, there is imminent danger of arresting the processes of both respiration and circulation.

CASE I.—A short time ago a lady came to my office with a very painful felon on her finger. I told her it must be opened, and that the operation would not hurt her any. Pouring out a mixture of equal parts of chloroform and ether, which I always use, on a handkerchief, I directed her to hold it to her mouth and inhale it freely, at the same time pinching her skin, asking if she felt it; the moment she said she did not, I laid open the felon to the bone. A moment after (she having been looking in the opposite direction in the meantime) asked me "when I was going to begin to act." I told her to look at her finger, and as she saw the blood running pretty freely, while the pain was wholly removed, she could hardly believe her own senses, as she had not lost consciousness for a moment, and declared she had not felt the slightest pain.

CASE II.—A few days since I was asked to pass a catheter in the case of Dr. T. of this village. I found he had been laboring under acute suppression of urine for twelve hours or more, accompanied with great pain and suffering from frequent unsuccessful attempts to pass a prostrate catheter himself. On making a careful attempt to introduce the instrument the Doctor was thrown into spasms, with violent pain and agony, the catheter not passing more than an inch or two within the urethra. I immediately sent for the chloroform mixture, and gave it in the same way as in the former case; and when the Doctor had no feeling from pinching, I passed the instrument without the slightest pain or difficulty—the patient not only, not *unconscious* of what was doing, but seeing and directing the operation all the while. He declared he felt not the slightest sensation during the passage of the instrument, nor while three pints of urine were being evacuated.

I could supplement these cases with scores of others, if necessary. I wonder that such a mistake as the above would be made at this day, and especially since the French physiologist, M. Flourens, demonstrated, several years since, that anæsthetics affected different portions of the brain successively; that sensation was affected before volition, and that the medulla oblongata was the last to feel its influence; and that when this was involved, destroying consciousness, the life of the patient was in imminent danger. I hold, that all the fatal cases from chloroform might have been prevented by following the simple method above pointed out: for it is demonstrated that death cannot happen where the article is slowly and cautiously administered, and great precaution observed.

CHARLES A. LEE, M. D.

Peekskill, Dec. 10th, 1870.

